# Identification and Functional Characterization of Acyl-ACP Thioesterases B (GhFatBs) Responsible for Palmitic Acid Accumulation in Cotton Seeds

**DOI:** 10.3390/ijms232112805

**Published:** 2022-10-24

**Authors:** Baoling Liu, Yan Sun, Xiaodan Wang, Jinai Xue, Jiping Wang, Xiaoyun Jia, Runzhi Li

**Affiliations:** 1 College of Agriculture, Shanxi Agricultural University, Jinzhong 030801, China; 2 College of Life Sciences, Shanxi Agricultural University, Jinzhong 030801, China

**Keywords:** cotton (*Gossypium hirsutum*), acyl-ACP thioesterase (FatBs), substrate specificity, palmitic acid (C16:0), expression pattern, heterologous expression assay

## Abstract

In spite of increasing use in the food industry, high relative levels of palmitic acid (C16:0) in cottonseed oil imposes harmful effects on human health when overconsumed in the diet. The limited understanding of the mechanism in controlling fatty acid composition has become a significant obstacle for breeding novel cotton varieties with high-quality oil. Fatty acyl–acyl carrier protein (ACP) thioesterase B (FatBs) are a group of enzymes which prefer to hydrolyze the thioester bond from saturated acyl-ACPs, thus playing key roles in controlling the accumulation of saturated fatty acids. However, FatB members and their roles in cotton are largely unknown. In this study, a genome-wide characterization of FatB members was performed in allotetraploid upland cotton, aiming to explore the GhFatBs responsible for high accumulations of C16:0 in cotton seeds. A total of 14 *GhFatB* genes with uneven distribution on chromosomes were identified from an upland cotton genome and grouped into seven subfamilies through phylogenetic analysis. The six key amino acid residues (Ala, Trys, Ile, Met, Arg and Try) responsible for substrate preference were identified in the N-terminal acyl binding pocket of GhFatBs. RNA-seq and qRT-PCR analysis revealed that the expression profiles of *GhFatB* genes varied in multiple cotton tissues, with eight *GhFatBs* (*GhA/D-FatB3*, *GhA/D-FatB4*, *GhA/D-FatB5*, and *GhA/D-FatB7*) having high expression levels in developing seeds. In particular, expression patterns of *GhA-FatB3* and *GhD-FatB4* were positively correlated with the dynamic accumulation of C16:0 during cotton seed development. Furthermore, heterologous overexpression assay of either *GhA-FatB3* or *GhD-FatB4* demonstrated that these two GhFatBs had a high substrate preference to 16:0-ACP, thus contributing greatly to the enrichment of palmitic acid in the tested tissues. Taken together, these findings increase our understanding on fatty acid accumulation and regulation mechanisms in plant seeds. *GhFatBs*, especially *GhA-FatB3* and *GhD-FatB4*, could be molecular targets for genetic modification to reduce palmitic acid content or to optimize fatty acid profiles in cotton and other oil crops required for the sustainable production of healthy edible oil.

## 1. Introduction

Cotton (*Gossypium* spp.), an important cash crop around the world, not only produces high-quality fiber, but also provides a large amount of vegetable oil [1]. Cottonseed oil produced by traditional upland cotton occupies about 25% of the seed weight, with large yields because of the wide planting area [2,3]. As an important resource of edible vegetable oil, cottonseed oil is commonly used in food products such as margarine, salad dressing foods and the deep frying of snack foods after removing toxic gossypol [1]. The major fatty acid components in cottonseed oil consist of palmitic acid (C16:0, ~25%), stearic acid (C18:0, ~2%), oleic acid (C18:1, ~15%), linoleic acid (C18:2, ~59%) and linolenic acid (C18:3, ~1.2%) [4,5]. Numerous studies have reported that excessive intake of saturated fatty acids, i.e., palmitic acid, can raise cholesterol levels in the blood to a level harmful to health, leading to the risk of hyperlipidemia, cardiovascular diseases and many of the so-called metabolic syndromes [6,7]. Based on U.S. Food and Drug Administration requirements, total saturates should be controlled within 7% in edible oil [8]. Thus, the unbalanced fatty acid ratio of cottonseed oil has great limits its application in health food production. Therefore, reducing saturated palmitic acid and improving the fatty acid composition of cottonseed oil to promote nutritional values has become an important target in cotton breeding.

In plant seeds, de novo fatty acid biosynthesis and the storage of triacylglycerol (TAG) mainly occur in the stroma of plastids and endoplasmic reticulum (ER), respectively. Initially, acetyl-CoA is catalyzed by carboxylation to form malonyl-CoA [9]. The next step is the transfer of malonate group from malonyl-CoA to soluble acyl carrier protein (ACP), and the newly-formed malonyl-ACP is then used as a two-carbon donor to form fatty acyl-ACPs after circular elongation of condensation, reduction, dehydration and second reduction reactions [10]. The fatty acyl-ACPs are terminated to release free fatty acids and *holo*-ACPs which can be reused for binding fatty acyls. Subsequently, the free fatty acids are transported out of plastids and into cytoplasm to be re-esterified to form fatty acyl-CoA pool. Finally, the acyl groups from acyl-CoAs are catalyzed to be incorporated into glycerol 3-phosphate and eventually to produce TAG stored in ER by a series of acyltransferases including GPAT (Glycerol-3-phosphate acyltransferase), LPAT (Lysophosphatidic acid acyltransferase), DGAT (Diacylglycerol acyltransferase) or PDAT (Phospholipid:diacylglycerol acyltransferase).

Fatty acyl–acyl carrier protein thioesterases (Fats, EC 3.1.2.14) are intraplastidial enzymes that terminate the process of de novo fatty acid elongation by hydrolyzing the thioester bond from acyl-ACPs, playing a crucial role in determining the chain length and saturation degree of fatty acids, and the composition of fatty acids in various tissues/organs of higher plants [11,12]. Different types of Fats can selectively catalyze different acyl-ACPs, resulting in different kinds of fatty acids being exported into the cytosol [13]. According to their amino acid sequences and preference for substrates, plant Fats can be further classified into two families, FatA and FatB [14]. Generally, FatAs prefer unsaturated 18:1-ACP and have low activity for 16:1-ACP [14,15]. FatBs mainly select saturated acyl-ACPs as substrates, with a broad range of selectivity from 8:0-ACP to 18:0-ACP [13,16]. For instance, UcFatB1 from *Umbellularia californica* specifically selects 12:0-ACP, and its overexpression in *Brassica napus* seeds significantly increased lauric acid (C12:0) level [16,17]. ChFatB1 from *Cuphea hookeriana* exhibits broad activities from 14:0-ACP to 18:0-ACP despite of strong preference for 16:0-ACP [14], whereas ChFatB2 highly selects 8:0-ACP and 10:0-ACP [18]. In most plants, FatBs show stronger substrate specificity for 16:0-ACP over 18:0-ACP. However, some FatBs had a half-to-half preference for both 16:0- and 18:0-ACP or higher selectivity for 18:0-ACP than 16:0-ACPs. For example, knocking out the *AtFatB* gene in *A. thaliana* resulted in a decrease in both palmitic acid and stearate by about 40~50% in seeds [19,20]. *MlFatB* from *Madhuca longifolia* resulted in much higher levels of C18:0 than C16:0 when heterologously expressed in seeds of *Brassica juncea* [21]. The *Arabidopsis thaliana* seeds overexpressing *EgFatB1* from *Elaeis guineensis* noticeably increased C16:0 accumulation from the original ~8% to ~33% in the transgenics [22]. Overexpression of *JcFatB* from *Jatropha curcas* in Arabidopsis increased higher levels of saturated fatty acids such as C16:0, C18:0, C20:0 and C21:0 in the transgenic seeds compared to the wild-type plants [20]. Collectively, the substrate preference of thioesterases is the key factor in determining the fatty acid composition of storage lipids in plants, and thioesterases are increasingly becoming the excellent target to be considered in the genetic improvement of fatty acid profiles in seed oils.

In the conventional cottonseed oil, palmitic acid (C16:0) makes up approximately 25% of the total fatty acids, indicating that a large pool of palmitoyl-ACP are formed in developing cotton seeds, and in particular, some cotton FatB enzymes may have strong substrate preference for palmitoyl-ACP. It might be the FatBs that contribute greatly to the high level of palmitic acid remaining in the final cottonseed oil. However, such kinds of FatBs and their acyl selection mechanism in cotton has not been thoroughly elucidated to date.

In this study, we performed the genome-wide characterization of GhFatB family members in upland cotton and a comparative analysis with other three cotton species (*G. arboreum*, *G. raimondii*, and *G. barbadense*), aiming to identify the GhFatBs responsible for the high accumulation of palmitic acid in cotton seeds. The key amino acids in the catalytic activity center were identified for GhFatBs. RNA-seq and qRT-PCR were employed to reveal the temporal and spatial expression patterns of *GhFatB* genes in various tissues/organs including different stages of developing cotton seeds. Furthermore, we investigated the dynamic changes of fatty acid components in the developing seeds of an upland cotton cultivar Zhongmian 21 and the association analysis of major fatty acid accumulation and the candidate *GhFatB* gene expressions in cottonseeds. Finally, two target genes, *GhA-FatB3* and *GhD-FatB4*, were cloned and consequently overexpressed in tobacco (*Nicotiana benthamiana*) to examine their functions in the biosynthesis of fatty acids, especially palmitic acid accumulation. Overall, the present findings lay new insights to deepen our understanding of the fatty acid metabolic network and regulation mechanism in plants. *GhA-FatB3* and *GhD-FatB4* can be used as the target genes for optimizing fatty acid composition in cottonseed oil or other oil crops to meet different nutrition and food requirements.

## 2. Results

### 2.1. Dynamic Profiles of Fatty Acids during the Development of Cotton Seeds

**Figure 1:**
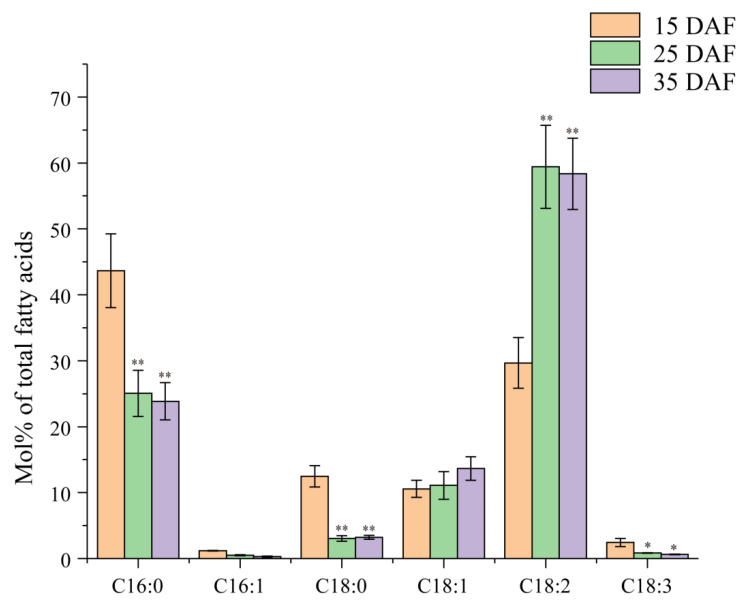
Fatty acid profiles of cotton seeds at three development stages (15DAF, 25DAF and 35DAF). Fatty acid level (mol%) was presented as a percentage of all fatty acids. Values are the mean ± SE of six biological duplicates. “*” and “**” represent statistically significant differences (*p* < 0.05 and *p* < 0.01) between the fatty acid level in the 15 DAF and the value in the 25 or 35 DAF seeds, based on two-tailed Student’s *t* tests.

To investigate the dynamic changes of fatty acid (FA) profiles during cotton seed development, the contents of FA components were measured by GC analysis for the developing cotton seeds from three stages denoted as 15 DAF (early stage), 25 DAF (middle stage) and 35 DAF (late stage). As shown in Figure 1, different accumulation patterns were detected for six major FAs during seed development. The most obvious difference is the content of palmitic acid (C16:0), stearic acid (C18:0) and linoleic acid (C18:2). Palmitic acid (C16:0) rapidly accumulated in the early stages and peaked at 15 DAF (43.65 mol% of total FAs), followed by a large reduction to 23.85 mol% at the late stage of 35 DAF (Figure 1). Similarly, the C18:0 content also noticeably decreased from the highest level with 12.47 mol% at the early stage to 3.23 mol% at the late stage (Figure 1). On the contrary, the content of linoleic acid (C18:2) displayed an overall increasing trend from initial 29.67 mol% at the early stage up to the peak of 59.42 mol% at the middle stage and the stable level of 58.35 mol% at the late stage of 35 DAF, thus becoming the most abundant fatty acid in mature cotton seeds (Figure 1). In addition, palmitoleic acid (C16:1) and linolenic acid (C18:3) had relatively small changes during cotton seed development. In short, the most significant changes of FA profiles occurred at the early and middle stages of cotton seed development, suggesting that the corresponding enzymes related to fatty acid synthesis and the accumulation function differed during the seed development.

### 2.2. Genome-Wide Identification of FatB Family Members from Four Cotton Species

To explore cotton FatB family members, AtFatB protein sequences were used as the queries to search against the four cotton species genomes, including allotetraploid *G*. *hirsutum*, and *G. barbadense*, as well as diploid *G. arboreum* and *G. raimondii*, respectively. According to BLAST results (Table 1), a total of 14 GhFatB members were identified from upland cotton (*G. hirsutum*), and the same number of GbFatBs was also detected from *G. barbadense* (Table S1). However, seven FatB members denoted as GaFatBs and GrFatBs were characterized from each of diploid *G. arboreum* and *G. raimondii* (Table S1). All the conserved domains of these putative FatB proteins were further identified by bioinformatics analysis using SMART, Pfam and CDD website, revealing that all those cotton FatB proteins contain the integrated sequences and a typical thioesterase domain. According to the chromosome position of A_t_/D_t_ sub-genomes, the upland cotton FatBs were renamed as GhA-FatB1 to GhA-FatB7 and GhD-FatB1 to GhD-FatB7 (Table 1). The protein lengths and relative molecular weights of GhFatB proteins ranged between 392~412 aa and 44.56~48.21 kD, respectively. However, the theoretical pI showed a wide fluctuation ranging from 5.76 to 9.26. All of the physical and chemical properties of GhFatBs were highly similar with FatBs from other three cotton species, thus again confirming the close genetic relationship between the A_t_ and D_t_ subgenome among these four cotton species. In addition, 12 of 14 GhFatB proteins had a hydrophobic region with a length of 14 aa in the N-terminal end, and this fragment (namely transit peptide) was probably used for transmembrane transport from cytoplasm to plastid (Table 1) [23]. As for GhA-FatB2 and GhD-FatB2, the hydrophobic region is located behind the hydrophilic region, suggesting that these two GhFatBs may have different structures.

### 2.3. Gene Structure, Conserved Domain and Phylogenetic Analysis of GhFatBs

**Figure 2:**
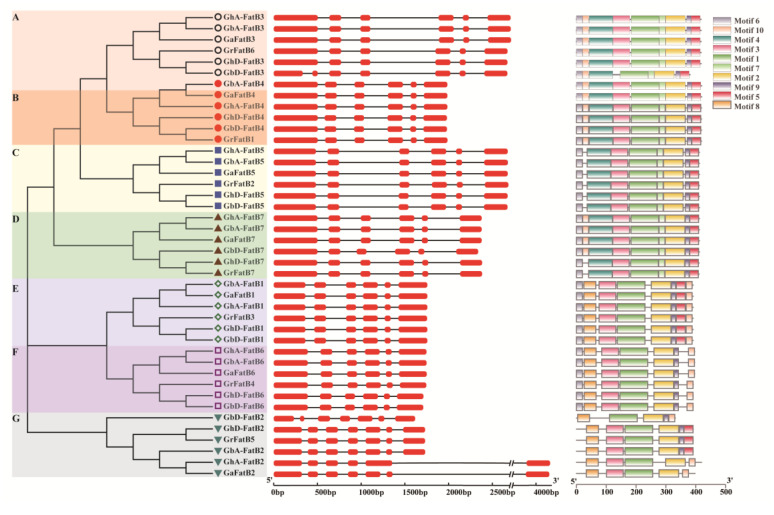
Gene structure and conserved motifs of FatBs in four cotton species (*G. arboreum*, *G. raimondii*, *G. barbadense* and *G. hirsutum*). “A~G” represent different FatB subfamilies. Blue blocks represent GhFatBs of *G. hirsutum*. Gene structure and conserved motifs are predicted by an online website of GSDS (http://gsds.cbi.pku.edu.cn/) (accessed on 12 October 2021) and MEME (http://meme-suite.org/index.html) (accessed on 12 October 2021), respectively. The exons and introns regions are displayed by red boxes and block lines, respectively. Motif 1, 2 and 3 are displayed with amino acid sequences. Each color represents an amino acid. The size of the letter represents the conservation of the amino acid at the corresponding position. The larger the letter, the stronger the conservation.

To analyze the evolutionary relationships of cotton FatBs, a phylogenetic tree was constructed using full-length amino acid sequences of all 42 cotton FatBs. As shown in Figure 2, cotton FatB proteins were classified into seven subgroups (namely A, B, C, D, E, F, and G). In each subgroup, two GhFatBs clustered together with two GbFatBs, one member from both GrFatBs and GaFatBs (Figure 2). Cotton *FatB* members in the same subgroup were also distributed at the same number chromosomes from the A_t_ or D_t_ genome (Figure 2). In addition, the gene structure analysis showed that all cotton *FatB* members contained six exons and five introns with different lengths except for subgroup G in which the exon numbers were seven, with one exception of *GhA-FatB2* having six exons. This finding suggests that the intron/exon structure is relatively conserved across different subgroups.

To better understand the structural evolution of GhFatBs, their conserved motifs were analyzed by the MEME-Suite website, showing that the common conserved motifs existed in the structurally similar members within the same subgroup. Total of 10 motifis were identified in all the 42 cotton FatBs (Figure 2), with different numbers of motifs across these subgroups. For instance, the motif numbers of GhFatB were 9 in both A and B groups while 8 motifs were detected in the C, D, and E groups, and 7 motifs in the F group, respectively (Figure 2). In group G, only 5 or 6 motifs were identified because of the sequence difference that existed in the six cotton FatB members.

Remarkably, all GhFatB members had the three signature motifs, namely Motif 1, Motif 2 and Motif 3, respectively (Figure 2), which are necessary for the function of all GhFatBs. The sequences of Motif 1 and Motif 3 constituted the substrate recognition center in the N-terminal of FatB enzymes and were crucial to determine the fatty acid profiles accumulated in plants. The Motif 1 and 2 sequences of FatB enzymes with substrate activities for short or medium chain FAs are different from those with substrate preferences for palmitic or stearic acids (Figure S1). In addition, the sequence in Motif 2 shaped the catalytic center of the FatB enzyme, thus characterizing the universality of FatB enzymes in catalytic activity. These results indicated that the *GhFatB* gene members were highly evolutionarily conserved despite a few differences being detected. The evolutionary relationship of these four cotton species revealed by the analyses above is in agreement with that described previously by Zhang et al. [24], showing that the genome of tetraploid *G. hirsutum* (A_t_A_t_D_t_D_t_) evolved from the diploid *G. raimondii* (D_t_D_t_) and *G. arboreum* (A_t_A_t_), whereas the former (D_t_D_t_) was the ancestral genome of the later (A_t_A_t_).

### 2.4. Multiple Sequence Alignments and 3D Structural Modelling of GhFatBs

To further characterize the sequence and structural features of GhFatBs, multiple sequence alignments and three-dimensional structure modelling were performed with the templates of 16:0-specific AtFatB/ZmFatB and 12:0-specific UcFatB examined by X-ray diffraction assays [23,25]. High similarity was detected between GhFatBs and the templates, showing that all GhFatBs contained two independent N-terminal and C-terminal hotdog domains (Figure S2). However, the intermediate region of the C- and N-terminal end showed as being poorly conserved, with it being linked by an intervening α3 helix.

**Figure 3:**
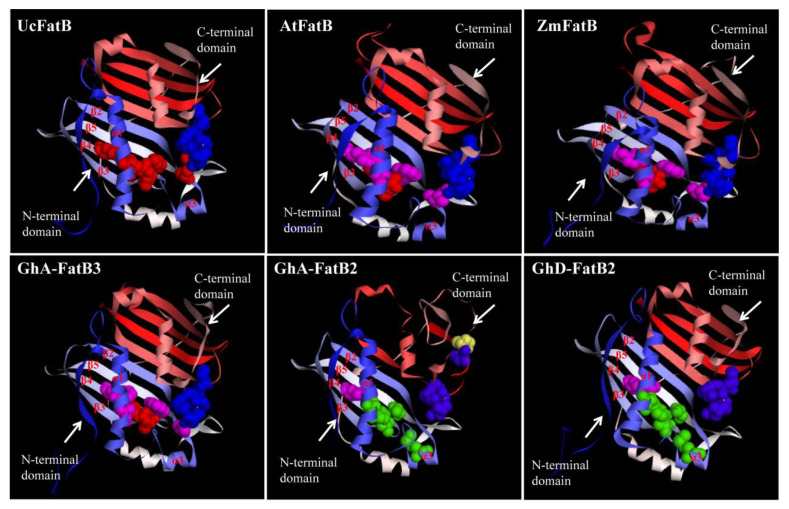
Three-dimensional structure models of GhFatB protein monomers. All 3D structures were predicted on Swiss-Model (https://swissmodel.expasy.org/) (accessed on 12 October 2021) with UcFatB (AAA34215.1) as the template. Files of 3D structure of GhFatBs were displayed in Discovery Studio 4.1 software. Blue bolls show the AAs affect enzyme activities in the C-terminal domain, whereas yellow bolls represent the varied AAs different from that of UcFatB. Red bolls represent the AAs as being the same as the UcFatB in N-terminal domain. Pink bolls show the variant AAs different from UcFatB while green bolls show the additional residues different from UcFatB, AtFatB, and ZmFatB in the domain.

The 3D structure modellings showed that GhFatB enzymes (e.g., GhA-FatB3) were homodimers and fitted well with the structural details of the templates (Figure 3). Each subunit contained a papain-like catalytic triad and an active site arranged by a hotdog mainly consisted of β sheets and α helixes except for GhA-FatB2 with uncanonical structure (Figure 3 and Figure S2). The C-terminal domain of GhFatBs had strong homology and contained the five key residues (Asp, Asn, His, Glu and Cys) essential for catalysis in the active center of FatB enzymes (Table 2). These amino acid residues were located in random coils structurally close to the N-terminal and constituted the uncanonical Asp-His-Glu catalytic network. Nevertheless, such analysis of GhFatB sequences and the models of the tertiary structure displayed some differences in N-terminal domains corresponding to the binding cavity relative to UcFatB. The six key amino acids (AAs) were identified in this binding cavity of GhFatBs (Table 2, Figure 3). These six AAs were located on β2, β3, β4 and β5 sheets of GhFatB enzymes, determining the fatty acyl into the substrate binding cavity along with α1 helix in the center of the β sheets (Figure 3). For 12 GhFatB members, all the six residues were the same as in the 16:0-specific AtFatB and ZmFatB, whereas the four residues (Table 2, Blue area: Ala, Trys, Ile and Try) were different from the 12:0-specific UcFatB (T137, S219, M163 and F201), suggesting that these four AA residues have an important function in the substrate selectivity of GhFatB for C16:0-ACP such as in the cases of AtFatB/ZmFatB. When a FatB enzyme performs the catalytic function in the form of a homodimer, these key AAs can form the substrate recognition center to control the kinds of fatty acyl-ACPs into the cavity by the size of their side chains. Finally, the binding position of the carbon chain of fatty acyl with ACP was adjusted to the enzyme activity center and was then hydrolyzed to form free fatty acids by the FatB enzyme. In addition, five of the six key amino acids in the N-terminal domain of GhA-FatB2 and GhD-FatB2 were different from the other GhFatBs and the templates tested, thus suggesting that these two GhFatBs may have different substrate selectivities or catalytic activities from other GhFatBs.

### 2.5. Tissue-Specific Expression Patterns of GhFatB Genes in Upland Cotton 

**Figure 4:**
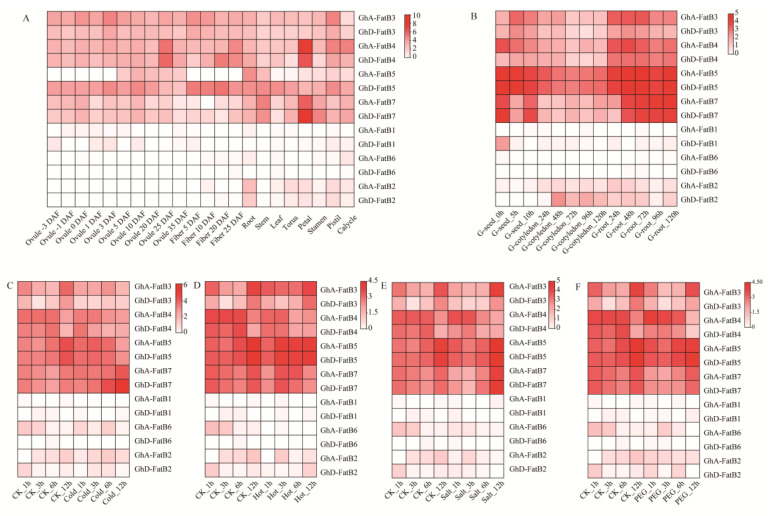
Transcriptional expression patterns of 14 *GhFatB* genes in various cotton tissues. The mRNA abundance was measured by the FPKM value (Fragments per kilobase of transcript per million mapped reads). The high value of FPKM represents the high expression level. The darkness of red color indicates the expression level. (**A**) Expression levels of 14 *GhFatB* genes in various cotton tissues including developing ovules (−3, −1, 0, 1, 3, 5, 10, 20, 25 and 35 DAF), developing fibers (5, 10, 20 and 25 DAF), vegetable tissues (root, stem, and leaf) and flower tissues (torus, petal, stamen, pistil, and calycle). (**B**) Expression levels of 14 *GhFatB* genes in various tissues of cotton seed germination including germinating seeds (0, 5, and 10 h), cotyledon (24, 48, 72, 96, and 120 h) and roots (24, 48, 72, 96, and 120 h). (**C**–**F**) Expression levels of 14 *GhFatB* genes in cotton leaves treated with cold stress (**C**), hot stress (**D**), salt stress (**E**) and drought stress (**F**), respectively. All the RNA-seq data used here were obtained from the Cottonfgd database (https://cottonfgd.org/) (accessed on 6 October 2021).

In order to investigate the potential functions of upland cotton *GhFatB* genes, their transcription patterns were examined in multiple tissues/organs including developing ovules and fibers, germinating seeds, as well as the abiotically stressed leaves using the transcriptomic data of upland cotton extracted from the Cottonfgd database (https://cottonfgd.org/) (accessed on 6 October 2021). As shown in Figure 4, the expression heatmap of eight *GhFatB* genes (*GhA/D-FatB3*, *GhA/D-FatB4*, *GhA/D-FatB5*, and *GhA/D-FatB7*) exhibited relatively high expression in almost all vegetative tissues (root, stem and leaves), reproductive tissues (torus, petal, stamen, pistil and calycle), developing seeds (ovules and fibers) and germinated tissues (seeds and roots), despite the differences in their expression abundance (Figure 4A,B), suggesting that these *GhFatBs* function crucially in fatty acid accumulation in those tissues/organs. Of them, *GhA/D-FatB3* and *GhA/D-FatB4* had higher expression levels than other *GhFatBs* in seeds and at the early and late stages of seed development, respectively (Figure 4A). Moreover, the expression abundance of these eight *GhFatB* genes was also stable and not affected by cold, hot, salt and drought stresses (Figure 4C–F). However, the six other *GhFatB* genes (*GhA/D-FatB1*, *GhA/D-FatB6*, and *GhA/D-FatB2*) had low mRNA abundance (FPKM ≤ 2) in all tested tissues, suggesting that they may be not involved in cotton responses to abiotic stresses. However, they might be induced by biotic stresses such as pathogen infections, revealed by an Arabidopsis disease resistance study (Zhao et al., 2022). Arabidopsis Acyl Carrier Protein 1 (ACP1), a small essential protein functioned as a carrier of the acyl intermediates in the fatty acid synthesis pathway, has a low expression in leaves, but can be induced and play an important role in plant immunity to bacterial pathogen infection.

**Figure 5:**
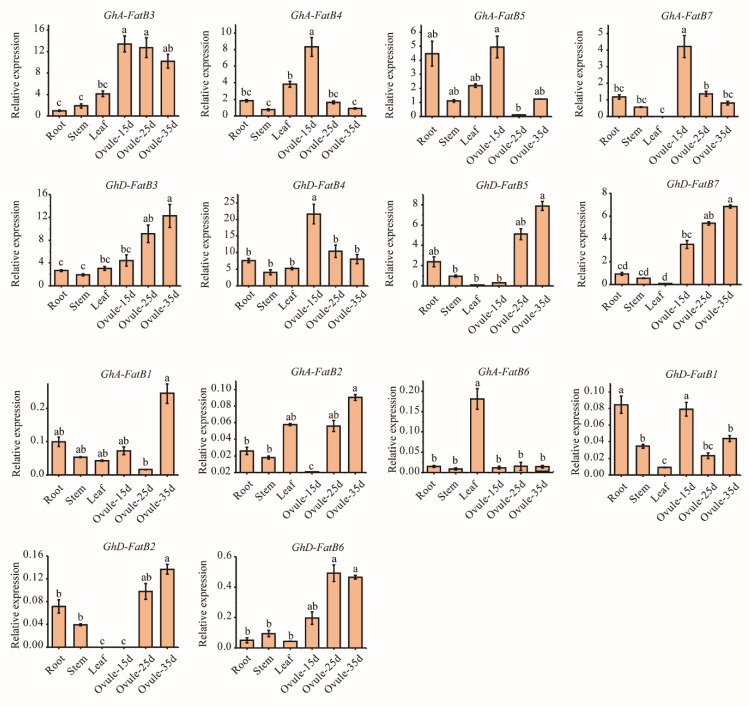
Expression patterns of 14 *GhFatB* genes in various cotton tissues examined by qRT-PCR analysis. The method of 2^−Δcq^ was used in this analysis. Bar charts represent the relative expression levels of *GhFatB* genes normalized by the internal control genes *GhHistone3* and *GhEF-1α* measured by qRT-PCR with three biological samples for each tissue. “Ovu-15d, 25d and 35d” represent the developing ovules at 15, 25 and 35 days after flowering, respectively. Histograms with different small letters are significantly different at 0.05 level by Tukey’s test.

To further verify the expression patterns of *GhFatB* genes, qRT-qPCR analysis was employed to examine the transcript abundance in roots, stems, leaves and developing ovules of upland cotton (15, 25 and 35 DAF). As shown in Figure 5, eight *GhFatB* genes (*GhA/D-FatB3*, *GhA/D-FatB4*, *GhA/D-FatB5* and *GhA/D-FatB7*) were highly expressed in all tested tissues compared with the six other *GhFatBs* (*GhA/D-FatB1*, *GhA/D-FatB2* and *GhA/D-FatB6*). Such qRT-qPCR data were consistent with the expression profiles of those genes revealed by transcriptome analysis (Figure 4A). Furthermore, these eight *GhFatBs* displayed higher expression levels in the developing ovules than in the vegetative tissues, especially high expression in the early-developing ovules (15 DAF), such as *GhA-FatB3* and *GhD-FatB4*. Notably, five out of the eight genes (*GhA-FatB3*, *GhA-FatB4*, *GhD-FatB4*, *GhA-FatB5* and *GhA-FatB7*) were highly expressed in ovules with a peak in the early stage (15 DAF), followed by slightly reduced expression levels in the middle and late stages of ovule development (Figure 5). However, along with ovule development, *GhD-FatB3*, *GhD-FatB5* and *GhD-FatB7* showed a gradually increasing expression pattern with a peak at the late stage of ovule development (35 DAF) (Figure 5). Overall, compared with vegetative organs, most of the *GhFatB* genes expressed highly in seeds, with much higher levels of *GhA-FatB3* and *GhD-FatB4* in the early stage of seed development, followed by higher levels of *GhD-FatB3*, *GhD-FatB5* and *GhD-FatB7* in the late stage of seed development. This expression profiling indicates that *GhA-FatB3* and *GhD-FatB4* may be responsible for the highest accumulation of C16:0 in the early stage of developing seeds.

**Figure 6:**
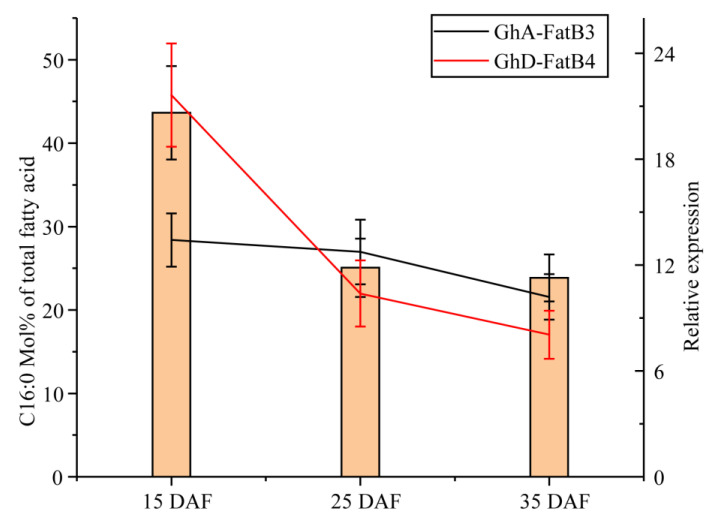
The correlation of the expression of *GhA-FatB3* and *GhD-FatB4* with molar percentage of C16:0 in the developing cotton seeds. The columns show the C16:0 content (mol% of total fatty acids) and lines for the relative expressions of the target genes (Black for *GhA-FatB3*, Red for *GhD-FatB4*). Six biological replicates were analyzed for all samples.

Coincidentally, the dynamic expression of *GhA-FatB3* and *GhD-FatB4* in ovules showed a positive association with palmitic acid (C16:0) levels in the developing cottonseeds (Figure 6). Furthermore, the linear regression coefficient was significant between C16:0 content and *GhD-FatB4* expression with *r* = 0.992. *GhA-FatB3* expression and palmitic acid accumulation also displayed a positive correlation with *r* = 0.690. This association analysis again evidenced that GhA-FatB3 and GhD-FatB4 probably contribute vitally to the biosynthesis and the accumulation of C16:0 in cotton seeds.

### 2.6. Fatty Acid Profiles of Leaves Transiently Expressing GhFatB Genes

**Figure 7:**
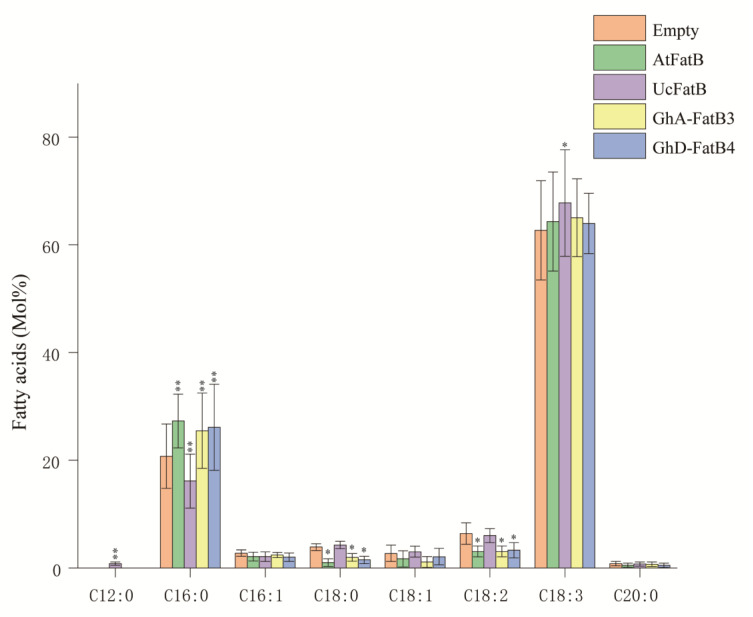
Fatty acid profiles in the *N. benthamiana* leaves transiently expressing *GhA-FatB3*, *GhD-FatB4*, *AtFatB* and *UcFatB*, respectively. Data are means of mol% ± SE with six biological replicates. “*” and “**” represent statistically significant differences (*p* < 0.05 and *p* < 0.01) based on two-tailed Student’s *t* tests compared with the negative control (empty vector).

To further identify the catalytic activity and substrate preference of GhFatBs, we selected GhA-FatB3 and GhD-FatB4 as the targets based on the analysis above. The recombinant plasmids containing *GhA-FatB3* or *GhD-FatB4* genes were transiently expressed in *N. benthamiana* leaves by *Agrobacterium*, respectively. Recombinant plasmids of 12:0-specific *UcFatB* or 16:0-specific *AtFatB* were selected as the positive controls to be transiently expressed in the leaves, respectively, because these two genes have been thoroughly characterized for their substrate specificities [13,25]. An empty vector was used as the negative control. Fatty acid profiles were examined for the tobacco leaves transiently expressing the target genes. As shown in Figure 7, the overexpression of *GhA-FatB3* or *GhD-FatB4* resulted in an increased level of C16:0, with 4.73 mol% and 5.36 mol% in the leaves, respectively. Meanwhile, contents of C18:0, C18:1 and C18:2 decreased slightly in the infected leaves compared to the empty-vector control leaves (Figure 7). As the positive control gene, overexpression of *AtFatB* also led to an obvious enhancement of C16:0 accumulation, with 6.55 mol% in the infected leaves. Unlike 16:0-specific FatBs, the overexpression of *UcFatB* caused new fatty acid (C12:0) synthesis up to a small amount (0.83 mol%) and also a decrease in the C16:0 level by 4.63 mol% (Figure 7). The consistent fatty acid profiles detected in the tobacco leaves transiently expressing *GhA-FatB3*, *GhD-FatB4* and *AtFatB*, respectively, revealed that both GhA-FatB3 and GhD-FatB4 had a high substrate specificity for 16:0-ACP, thus leading to the high-level accumulation of palmitic acid in cottonseed oil.

## 3. Discussion

The quality and nutritional value of plant oils are largely defined by their fatty acid compositions. As the sixth largest vegetable oil source in the world, cottonseed oil contains 25% palmitic acid as the second highest fatty acid following 59% linoleic acid. In general, palmitic acid-enriched vegetable oils including cottonseed oil are widely present in our diet system. However, overconsumption or long-term consumption of an oil rich in saturated C16:0 causes a negative impact on human health. Therefore, there is great practical value in effectively reducing the content of C16:0 in cottonseed to improve the nutritional quality of cottonseed oil. Understanding fatty acid and oil metabolism is the prerequisite of improving oil quality and increasing oil yield. Although the basic biochemical pathways of fatty acid biosynthesis and storage oil production in plants have been well documented, the factors regulating fatty acid profiles and controlling the total oil content in cotton and other oilseed crops remain to be elucidated.

For de novo biosynthesis of fatty acids in plant plastid, acyl-ACP thioesterase (Fat) can terminate the continuous extension of acyl-ACP and release free fatty acids which are mostly channeled to form TAGs through a series of enzymatic reactions. The known studies revealed that Fat enzyme was a major contributing factor in determining the carbon chain length of fatty acids through their substrate specificity. Among the two families of Fat enzymes (e.g., FatA and FatB), FatA shows extremely high substrate preference to unsaturated acyl-ACPs, whereas FatB mainly hydrolyzes saturated acyl-ACPs with a chain length of C8 to C18. In most plant tissues, the products of the typical FatB enzymes are mainly C18:0 and C16:0. For example, both peanut AhFatB and MbFatB from *Madhuca butyracea* displayed the specificity for C16:0 [26], while soybean GmFatB specially selected substrate C18:0 [8]. *C. longepaniculatum* ClFatB1 showed more preference for C16:0 over C18:0 [27]. In addition, several FatBs from high accumulators of short/medium chain FAs (C8~C14) have high substrate specificity to short- or medium-chain saturated acyl-ACPs, such as C8:0/10:0-specific CpuFatB from *Cuphea pulcherrima* [28], and 12:0-specific UcFatB from *Umbellularia californica* [29]. However, reports of FatB function are still relatively limited in cotton. In particulr, FatB members responsible for the high accumulation of C16:0 in cotton seeds have not been not identified yet.

In this study, we carried out a genome-wide characterization of cotton FatBs, and total of 14 FatB members were identified in allotetraploid cultivated upland cotton (A_t_A_t_D_t_D_t_) and island cotton (A_t_A_t_D_t_D_t_), respectively (Table 1 and Table S1). The numbers of FatBs in each allotetraploid cotton species were twice as many as in each of diploid cotton species *G. arboreum* (A_t_A_t_) and *G. raimondii* (D_t_D_t_). Alignment analysis (Figure S2) showed that the coding sequences of *GhFatB* genes from the A_t_ subgenome were almost identical to the D_t_ subgenome except for a few base differences, such as gene pairs of *GhA/D-FatB3*, *GhA/D-FatB4* and so on. Furthermore, the high similarity was also detected in cotton *FatB* gene structures, conserved protein domains and evolutionary relationships (Table 1, Figure 1). These results again demonstrated a very good matching relationship that the ancestral genome of allotetraploid *G. hirsutum* (A_t_A_t_D_t_D_t_) came from diploid *G. arboreum* (A_t_A_t_) and *G. raimondii* (D_t_D_t_), while D_t_D_t_ genome also originally evolved from A_t_A_t_ genome [30,31].

More importantly, protein sequence alignment (Table 2) and 3D structure modelling (Figure 3) revealed that the five key amino acid residues (Asp, Asn, His, Glu and Cys) essential for catalysis in the C-terminal catalytic center (within one hotdog domain) were almost identical in 14 GhFatBs and the 3 reference FatBs (AtFatB, ZmFatB and UcFatBs). Moreover, the six key amino acid residues (Ala, Trys, Ile, Met, Arg and Try) responsible for substrate recognition were identified in the N-terminal acyl binding pocket (within the other hotdog domain) of 12 GhFatBs except for GhA-FatB2 and GhD-FatB2, which were the same as that in the 16:0-specific AtFatB/ and ZmFatB, but four of these AAs (Ala, Trys, Ile, and Try) different from that of the 12:0-specific UcFatB (Table 2, Figure 3). This sequence comparative analysis indicates that the 12 GhFatB enzymes may highly select 16:0-ACP as the catalytic substrate, and the four AAs might function much importantly in determining the cavity size for the substrate entering into the cavity of the FatB enzymes. These key AAs identified from GhFatBs provide the scientific reference for predicting the substrate specificity of FatBs which are yet to have functional characterization, and they also serve as the excellent targets for precise site mutation to obtain the customized structures of GhFatB enzymes so as to reduce C16:0 or alter the fatty acid composition in cotton or other oilseeds to produce the desired heath vegetable oils.

Expression profiling can provide insights into the putative function of genes and the regulatory mechanisms of biological events in specific growth and development phases. We examined the expression patterns of 14 *GhFatB* genes in multiple upland cotton tissues (Figure 4) using both RNA-seq and real-time RT-PCR data, revealing various expression patterns of these *GhFatB* genes in all cotton tissues tested (Figure 4 and Figure 5). The similar expression patterns were detected for a few pairs of *GhFatBs*, pointing to functional redundancy during cotton evolution, which could result in neofunctionalization and subfunctionalization within the cotton *FatB* gene family. Notably, of 14 *GhFatB* genes, eight members including *GhA/D-FatB3*, *GhA/D-FatB4*, *GhA/D-FatB5*, and *GhA/D-FatB7* exhibited relatively high expression in almost all the tissues tested, particularly in developing ovules, indicating that the eight GhFatBs play an important role in releasing free fatty acids to cytosol during cotton seed development. It should be noted that these eight *GhFatB* expressions were not significantly affected by abiotic stresses (Figure 4C–F), showing that they may rarely participate in abiotic stress defense. Furthermore, five of these eight *GhFatBs* (*GhA-FatB3*, *4*, *5*, *7* and *GhD-FatB4*) exhibited much higher levels of expressions in the early stage of seed development whereas the other three *GhFatBs* (*GhD-FatB3*, *5* and *7*) largely expressed in the late stage of seed development. In consistence with the findings, such high expression patterns in developing seeds were also reported for *RcFatB* (*Ricinus communis*) [32], *HaFatB* (*Helianthus annuus*) [33] and *CsFatB* (*Camelina sativa*) [34], thus showing a high coincidence between *FatB* expression and the dynamic accumulation of fatty acid profiles during seed development. As expected, the expression patterns of *GhA-FatB3* and *GhD-FatB4* were positively correlated with the accumulation of C16:0 during cotton seed development (Figure 6), thus evidencing that GhA-FatB3 and GhD-FatB4 may be the major contributors to the high-level synthesis of palmitic acid in developing cotton seeds. In regard to high levels of C18:2, and certain levels of C18:1, and C18:0 in mature cotton seeds, the *GhFatB* members with high expressions in the middle and late stages of seed development possibly contribute to this fatty acid synthesis and accumulation, cooperated with the functions of other related enzymes such as beta-ketoacyl-ACP synthase II (KASII), stearoyl-ACP desarurase (SAD), acyl-ACP thioesterase A (FatA) and fatty acid desaturase (FAD2), etc. Such speculation is also consistent with the fact that *GhSAD* and *GhFAD2* genes were highly expressed in the middle and late developing ovules of cotton [35,36]. Of course, other acyltransferases such as DGAT and PDAT also affected the fatty acid composition stored in TAG because they may specifically select these fatty acids to incorporate into TAGs [37]. In short, the biosynthesis and accumulation of fatty acids is an extremely complex system consisting of many enzymes functioning spatiotemporally. The detailed mechanism responsible for the dynamic of fatty acid synthesis and accumulation during cotton seed development needs further deep investigation. In addition to the abovementioned genes, there was the presence of six other GhFatB genes with low expression levels in different organs and under abiotic stress treatment. Nevertheless, we cannot rule out their possible roles associated with biotic defense responses. For example, Zhao et al. [38] identified an acyl carrier protein 1 gene (*ACP1*), a cofactor in Arabidopsis fatty acid biosynthesis, which has a low expression in leaves, but it can be induced and play an important role in plant immunity to bacterial pathogen infection.

Finally, *GhA-FatB3* and *GhD-FatB4* genes were heterologously expressed in the leaves of *N. benthamiana* to further verify their functions in promoting C16:0 synthesis and accumulation. Fatty acid profiling in the leaves (Figure 7) demonstrates that the C16:0 content was increased, but there was no significant change for C18:0 and C18:1 levels in the leaves, thus revealing that these two GhFatB enzymes had strong substrate specificity towards 16:0-ACP. Such 16:0-specific function is the same as AtFatB whose overexpression significantly increased C16:0 accumulation in the transgenic Arabidopsis seeds [13]. Previously, different substrate preferences were also identified for several FatBs derived from other plants by heterologous expression assay. For instance, MbFatB from *Madhuca butyracea* could promote the accumulation of C16:0 and C18:1, but had no effect on C18:0 [39]. MlFatB from *Madhuca longifolia* only had strong substrate specificity for C18:0 [21]. KpFatB prefered 18:0-/18:1-ACP over 16:0-ACP [40].

It should be noted that heterologous overexpression of *GhA-FatB3* or *GhD-FatB4* indeed increased C16:0 accumulation, but the enhanced level was limited. The other fatty acid profiles showed no significant changes. The similar situation was also observed in the *GmFatB1*-overexpressed plants [41]. Theoretically, complex factors mediate fatty acid synthesis and accumulation in plant seeds. In the transgenic leaves of *GhA-FatB3* or *GhD-FatB4*, the tobacco endogenous FatB members may be triggered to balance the accumulation of different fatty acids. From the perspective of the fatty acid synthesis pathway in plants, the “Source-Sink-Flow” pathway controls the fatty acid flux, proved by fatty acid metabolic assembly. For instance, the C10:0 level in camelina seeds was promoted from the initial 4 mol% in the wild-type up to 13.5 mol% in the transgenic lines co-expressing *CvFatB1* (*Cuphea viscosissima*) and *CpuDGAT1* (*Cuphea avigera* var. *pulcherrima*) genes, and a higher level (21.5 mol%) was obtained in the lines coordinated expressing *CvFatB1*, *CvLPAT2* and *CpuDGAT1* genes [42]. Significant alteration in fatty acid profiles can be achieved by enlarging the substrate pool and accelerating fatty acid transport. If we want to obtain the desired fatty acid profiles in the transgenic oil crops, further efforts are needed to simultaneously operate multiple genes in the fatty acid synthesis pathway. Thus, these *GhFatB* genes, particularly *GhA-FatB3* or *GhD-FatB4*, can be used as the promising targets to genetically modify fatty acid composition in cottonseed oil to improve oil quality and yield.

## 4. Materials and Methods

### 4.1. Plant Material

Healthy seeds of *G. hirsutum* cv. Zhongmian 21 were planted in Agricultural College Experimental Station at Shanxi Agricultural University, Taigu, China. Roots, stems and leaves were collected from 2-week-old seedlings. Petals were picked on the day of flowering. Developing ovules at 15, 25 and 35 days after flowering (DAF) were sampled from cotton bolls. All samples were instantly frozen in liquid nitrogen and stored at −80 ℃ for subsequent RNA extraction. Tobacco (*N. benthamiana*) was planted in an artificial incubator where the temperature, relative humidity and photoperiod were set as 26 ℃, 60%, and 14 h light/10 h dark, respectively [35,43]. Six-week-old seedlings were used for transient expression assays.

### 4.2. Gene Isolation, Gene Structure, Functional Domain and Phylogenetic Analysis

The genome sequences of four cotton species (*G. arboreum*, *G. raimondii*, *G. barbadense* and *G. hirsutum*) were retrieved from the Cotton Functional Genomics Database (https://cottonfgd.org/) (accessed on 6 October 2021) and cottongen (https://www.cottongen.org) (accessed on 6 October 2021). *AtFatB* gene sequences of Arabidopsis from TAIR website (https://www.Arabidopsis.org/) (accessed on 6 October 2021 ) were used as query to blast putative *FatB* genes in cotton database. All putative cotton FatB proteins were checked for the presence of FatB domain through CDD database (http://www.ncbi.nlm.nih.gov/Structure/cdd/wrpsb.cgi) (accessed on 6 October 2021) and SMART database (http://smart.Emblheidel-berg.de/) (accessed on 6 October 2021). *GhFatB* genes were renamed according to location orders from short to long arms of the chromosomes, respectively. The molecular weight and isoelectric point (pI) of GhFatB proteins were predicted by the Expasy-Protparam tool (https://web.expasy.org/protparam/) (accessed on 10 October 2021). TargetP 1.1 (http://www.cbs.dtu.dk/services/TargetP/) (accessed on 10 October 2021) and ProtComp 9.0 (http://www.softberry.com/berry.phtml) (accessed on 10 October 2021) were used to predict subcellular localization of GhFatB proteins.

The online website of GSDS (http://gsds.cbi.pku.edu.cn/) (accessed on 12 October 2021) was used to display exon/intron boundaries of *FatB* genes from four cotton species. The secondary structures of GhFatB proteins were predicted by SOPMA tool (https://npsa-prabi.ibcp.fr/cgi-bin/secpred_hnn.pl) (accessed on 12 October 2021). MEME Suite (http://meme-suite. org/index.html) (accessed on 12 October 2021) was employed to identify conserved motifs of FatB proteins of the four aforementioned cotton species, and the number and width of the motifs were set to 10 and 6~200, respectively. Further, all cotton FatB protein sequences were employed to construct a phylogenetic tree by MEGA 7.0 software with the Neighbor-Joining method and 1000 of bootstrap values.

### 4.3. Multiple Alignment and Three-Dimensional Model Analysis

C12:0-specific UcFatB (AAA34215.1) from *Umbellularia californica* and C16:0-specific AtFatB (CAA85388.1) from *A. thaliana* [13] as well as ZmFatB (AFW85914.1) from *Zea mays* were used as the templates and were obtained from NCBI (http://www.ncbi.nlm.nih.gov/) (accessed on 12 October 2021) [44]. All the protein sequences of cotton FatB and the templates were aligned by Gendoc software (http://www.nrbsc.org/downloads/, accessed on 12 October 2021) to find the basic structures and key amino acid residues which influence catalytic and substrate-specific properties in functional domain. Three-dimensional (3D) structures of GhFatBs were modelled by the online website SWISS-MODEL (https://swissmodel.expasy.org/) (accessed on 12 October 2021), where the crystal structure of UcFatB (PDB: 5X04A) was used as the template [25]. In addition, the key amino acids in the catalytic activity center were marked by Discovery Studio 4.1 software with default parameters.

### 4.4. RNA-Seq Analysis, RNA Isolation and qRT-PCR 

RNA-seq data of upland cotton (*G. hirsutum*) were obtained from the Cottonfgd database (https://cottonfgd.org/) (accessed on 6 October 2021). The mRNA abundance of *GhFatB* genes were measured by the FPKM value (Fragments per kilobase of transcript per million mapped reads) in various tissues, including vegetative tissues (root, stem and leaf), reproduction organs (torus, petal, stamen, pistil and calycle), developing seeds (ovules before flowering 1 and 3 days; ovules after flowering 0, 1, 3, 5, 10, 20, 25 and 35 days; fiber after flowering 5, 10, 20 and 25 days), geminated tissues (seeds of 0, 5 and 10 h; cotyledon and roots of 24, 48, 72, 96 and 120 h) and stressed tissues (roots of 1, 3, 6 and 12 h in control, cold, heat, salt and drought treatment). Finally, the resulting expression data of FPKM were used to generate heatmaps by R package (pheatmap: version 1.0.8).

To further investigate the expression patterns of *GhFatB* genes, total RNA from cotton ovules and vegetative tissues was extracted according to the protocol as described previously [35]. RNA was reversely transcribed into the first-strand cDNA by the PrimeScript™ RT reagent Kit for qPCR according to the manufacturer’s instructions (Takara, Inc., Dalian, China). qRT-PCR was performed on Biorad CFX96 (Bio-Rad, Hercules, CA, USA). The amplification parameters were performed following the protocol as previous described [35]. Fold changes were calculated with the method of 2^−ΔCq^ [45]. *GhEF-1α* (DQ174251) and *GhHistone3* (AF024716) were used as the internal controls for normalization [46,47]. Each sample was set three biological replicates. Gene-specific primers were designed based on cDNA sequences. All the primers used in this study were designed using the software premier 6.0 and are shown in Table S2.

### 4.5. Cloning of GhFatB Genes and Construction of Recombinant Expression Vectors

The cDNAs derived from the mixed developing ovules (15 and 25 DAF) were amplified to obtain the complete open reading frame (ORF) of the target genes (*GhA-FatB3* and *GhD-FatB4*) with cloning primers (Table S3). The 20 µL amplification solution contained cDNA (1.0 µL), 2 × Taq PCR Master Mix (10 µL), forward and reverse primers (2.0 µL in total), and nuclease-free ddH_2_O (7 µL) (ABM biotech., Zhenjiang, China). The PCR program was set up according to the previous protocol [47]. Then, the PCR products were successfully sequenced after a series of procedures including gel extraction, purification, cloning vector connection (pEASY-blunt Zero vector, TransGen Biotech, Beijing, China), transformation and screening of *Escherichia coli* strain (DH5α). Following that, the amplified fragment was digested with restriction enzymes and then constructed into a plant-expression vector of pCAMBIA1303 (CaMV 35S promoter, Invitrogen, Carlsbad, CA, USA) by T_4_ ligase to form a recombinant vector of Pro35S::GhA-FatB3 and Pro35S::GhD-FatB4. The individual ORF of *UcFatB* and *AtFatB* genes was artificially synthesized, and the recombinant vectors were constructed with the same methods above to form Pro35S::UcFatB and Pro35S::AtFatB. Finally, all positive recombinant vectors were separately transformed into *A. tumefaciens* (GV3101). The transgenic *A. tumefaciens* with an empty vector was used as the negative control. Primers related to vector construction are displayed in Table S3.

### 4.6. Transient Expression of GhFatB Genes in N. benthamiana

All stains of GV3101 containing the recombinant plasmids were separately cultured at 28 °C overnight and the cell pellets were collected by centrifugation when the value of OD_600_ was up to about 0.2. Before infiltration, the bacteria were resuspended by a suspension (OD_600_ ≈ 0.2) prepared with sterilized ddH_2_O containing acetosyringone (200 µmol/L), MgCl_2_ (10 mM/L) and MES (10 mM/L). Then, the six-week-old *N. benthamiana* leaves were infected with the method of Agrobacterium infiltration as previously described [35,48]. Lastly, the infection parts of the treated seedlings were freeze-dried for extraction of fatty acid methyl esters after five days of normal cultivation.

### 4.7. Extraction and Detection Analysis of Fatty Acid Methyl Ester

All the *N. benthamiana* samples and the developing cotton ovules (15, 25 and 35 DAF) were freeze-dried and grinded into powder. Total lipids were extracted by chloroform: methanol (2:1, *v*/*v*) and converted into fatty acid methyl esters (FAMEs) by 2.5% (*v*/*v*) sulfuric acid/methanol as previously described [21,35]. Before the lipid extraction, an appropriate amount of C17:0 TAG (Sigma-Aldrich, Saint Louis, MO, USA) was added as the internal standard into the sample. Then, FAMEs were analyzed by gas chromatograph with an Agilent equipment flame ionization detector as described previously [35]. Data were collected and calculated according to the retention time of fatty acid standard and peak area normalization of C17:0.

## 5. Conclusions

In this study, a total of 14 GhFatB members were identified from an upland cotton genome and classified into seven subgroups with similar protein structures and conserved domains. Notably, the six key amino acid residues (Ala, Trys, Ile, Met, Arg and Try) responsible for substrate specificity were identified in the N-terminal acyl binding pocket of 12 GhFatBs. Gene expression patterns of *GhFatBs* varied in multiple tissues but were unchanged by abiotic stress, with eight *GhFatBs* (*GhA/D-FatB3*, *GhA/D-FatB4*, *GhA/D-FatB5*, and *GhA/D-FatB7*) having high levels in developing ovules/seeds of cotton. In particular, expression profiles and heterologous overexpression assay of either *GhA-FatB3* or *GhD-FatB4* evidence that these two GhFatBs have a high substrate preference to 16:0-ACP and contribute greatly to the high accumulation of palmitic acid in cotton seeds. The present findings bring new insights into understanding FatB-mediated fatty acid biosynthesis and accumulation in developing cotton seeds. The identified GhA-FatB3 and GhD-FatB4 have a potential for genetic engineering to produce vegetable oils with low-level palmitic acid or desirable fatty acid profiles in cotton or other oilseeds for healthy food applications.

## Figures and Tables

**Table 1:** Basic information of upland cotton GhFatB members.

Gene ID.	Gene Name	Chromosome Location	Protein Length (aa)	Relative Molecular Weight (kD)	Theoretical pI	NCBI Accession	Hydrophobic Region (aa)
Gh_A03G1318	*GhA-FatB1*	A03:91082126..91083883	392	44.71	9.27	XP_012479545.1	1~13
Gh_A05G1069	*GhA-FatB2*	A05:10796095..10800280	420	48.21	8.65	XP_012440572.1	14~18, 20~22
Gh_A06G0514	*GhA-FatB3*	A06:10526649..10529358	418	46.17	7.65	XP_012451088.1	1~14
Gh_A07G0026	*GhA-FatB4*	A07:285980..287966	420	46.47	7.68	XP_012448973.1	1~14
Gh_A08G2137	*GhA-FatB5*	A08:102491658..102494334	413	45.65	6.42	XP_012477171.1	1~14
Gh_A12G1306	*GhA-FatB6*	A12:69335538..69337285	397	45.1	8.11	XP_012438233.1	1~14
Gh_A13G1750	*GhA-FatB7*	A13:76380863..76383241	413	46.01	8.53	XP_012461322.1	1~13
Gh_D02G1759	*GhD-FatB1*	D02:59968397..59970154	392	44.56	9.26	XP_012479545.1	1~14
Gh_D05G1218	*GhD-FatB2*	D05:10460475..10462206	392	45.34	8.78	XP_012440572.1	14~22
Gh_D06G0571	*GhD-FatB3*	D06:8969914..8972587	418	46.17	8.5	XP_012451088.1	1~14
Gh_D07G0034	*GhD-FatB4*	D07:343874..345855	420	46.46	7.07	XP_012448973.1	1~14
Gh_D08G2504	*GhD-FatB5*	D08:64827154..64829829	413	45.66	5.76	XP_012477171.1	1~14
Gh_D12G1429	*GhD-FatB6*	D12:44061277..44062989	392	44.72	8.94	XP_012438233.1	1~14
Gh_D13G2098	*GhD-FatB7*	D13:56456813..56459196	412	45.75	7.66	XM_016864037.1	1~13

**Table 2:** Key amino acids in the functional domain of various GhFatBs based on sequence alignment.

Protein Name	C-Terminal Catalytic Center					N-Terminal Acyl Binding Pocket					
UcFatB	D281	N283	H285	E319	C320	T137	S219	M163	M197	R199	F201
AtFatB	D313	N315	H317	E351	C352	A169	W251	I195	M229	R231	W233
ZmFatB	D324	N326	H328	E362	C363	A174	W256	I200	M234	R236	W238
GhA-FatB3	D315	N317	H319	E353	C354	A171	W253	I197	M231	R233	W235
GhD-FatB3	D315	N317	H319	E353	C354	A171	W253	I197	M231	R233	W235
GhA-FatB4	D315	N317	H319	E353	C354	A171	W253	I197	M231	R233	W235
GhD-FatB4	D315	N317	H319	E353	C354	A171	W253	I197	M231	R233	W235
GhA-FatB5	D308	N310	H312	E346	C347	A164	W246	I190	M224	R226	W228
GhD-FatB5	D308	N310	H312	E346	C347	A164	W246	I190	M224	R226	W228
GhA-FatB7	D314	N316	H318	E352	C353	A170	W251	I196	M229	R231	W233
GhD-FatB7	D313	N315	H317	E351	C352	A169	W251	I195	M229	R231	W233
GhA-FatB1	D267	N269	H271	E305	C306	A124	W204	I150	M182	R184	W186
GhD-FatB1	D267	N269	H271	E305	C306	A124	W204	I150	M182	R184	W186
GhA-FatB6	D276	N278	H280	E314	C315	A133	W215	I159	M193	R195	W197
GhD-FatB6	D276	N278	H280	E314	C315	A133	W215	I159	M193	R195	W197
GhA-FatB2	D315	N317	H319	E360	P361	S149	A232	V170	V210	F212	W214
GhD-FatB2	D293	N295	H297	E331	C332	S149	A232	V170	V210	F212	W214
Second structure	Random coil					α1 helix	β5 sheets	β2 sheets	β4 sheets	β4 sheets	β4 sheets

Note: 12:0-ACP specific UcFatB and 16:0-ACP specific AtFatB as well as ZmFatB are used as the references in multiple sequence alignment by Genedoc software. The GenBank or TAIR accession numbers of the template sequences were listed as the follows: UcFatB (AAA34215.1) from *U. californica*; AtFatB (CAA85388.1) from *A. thaliana* and ZmFatB (AFW85914.1) from *Z. mays*. Yellow and light blue colors represent the identical AAs and the divergent AAs compared to UcFatB, respectively. The purple color stands for AAs different from UcFatB, AtFatB and ZmFatB, respectively. Underlined parts show the variant AAs in the enzyme activity center of GhA-FatB2. The second structure of all FatBs was predicted using online tools (https://npsa-prabi.ibcp.fr/cgi-bin/secpred.hnn.pl) (accessed on 12 October 2021).

## Data Availability

Publicly available datasets were analyzed in this study. These data can be found here: https://cottonfgd.org/ (accessed on 6 October 2021); https://www.cottongen.org (accessed on 6 October 2021).

## Supplementary Materials

The supporting information can be downloaded at: https://www.mdpi.com/article/10.3390/ijms232112805/s1.
