# Supplementary material for: Identification and Functional Characterization of Acyl-ACP Thioesterases B (GhFatBs) Responsible for Palmitic Acid Accumulation in Cotton Seeds
Source: Int J Mol Sci. 2022 Oct 24;23(21):12805. doi: 10.3390/ijms232112805 (PMC9659231; doi:10.3390/ijms232112805)
Supplement: Supplementary file 1 [file ijms-23-12805-s001.zip › Supplementary Figures S1 and S2.pdf]

Number	Species	Name	Accession Number	Substrate Specificity
1	*Camelina sativa*	CsFatB1	AFQ60949.1	16:0
2	*Arabidopsis thaliana*	AtFatB	CAA85388. 1	16:0
3	*Cuphea avigera var. pulcherrima*	CpuFatB1	AGG79283	16:0
4	*U.californica*	UcFatB	Q41635.1	12:0
5	*Cuphea viscosissima*	CvFatB1	AEM72522	8:0
6	*Cuphea viscosissima*	CvFatB2	AEM72523	14:0
7	*Caltha palustris*	CpFatB2	AAC49180	14:0
8	*Cuphea hookeriana*	ChFatB2	AAC49269	8:0, 10:0
9	*Cuphea avigera var. pulcherrima*	CpuFatB3	AGG79285	8:0, 10:0

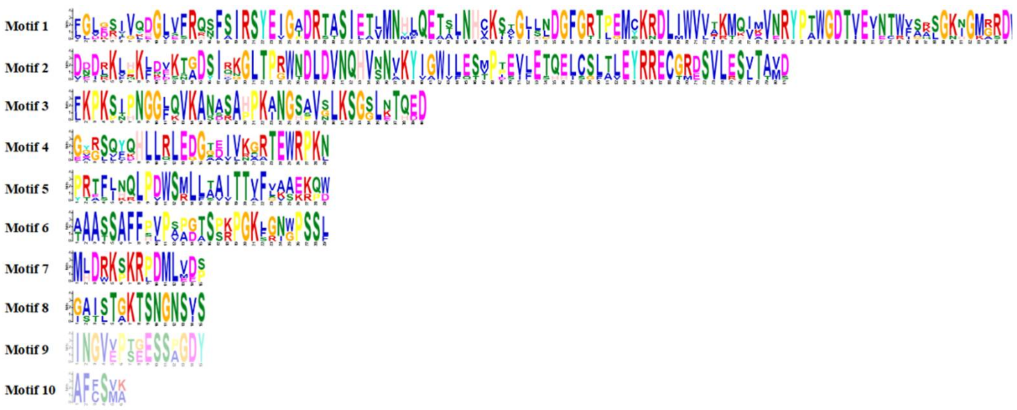

Supplementary Figure S1. Information and motifs of FatB members in species with short or medium fatty acid specificity predicted by MEME (http://meme-suite.org/index.html). Conserved animo acids in motifs are displayed with the size of the letter and the large size stands for strong conservation. Each color represents a type of amino acid.

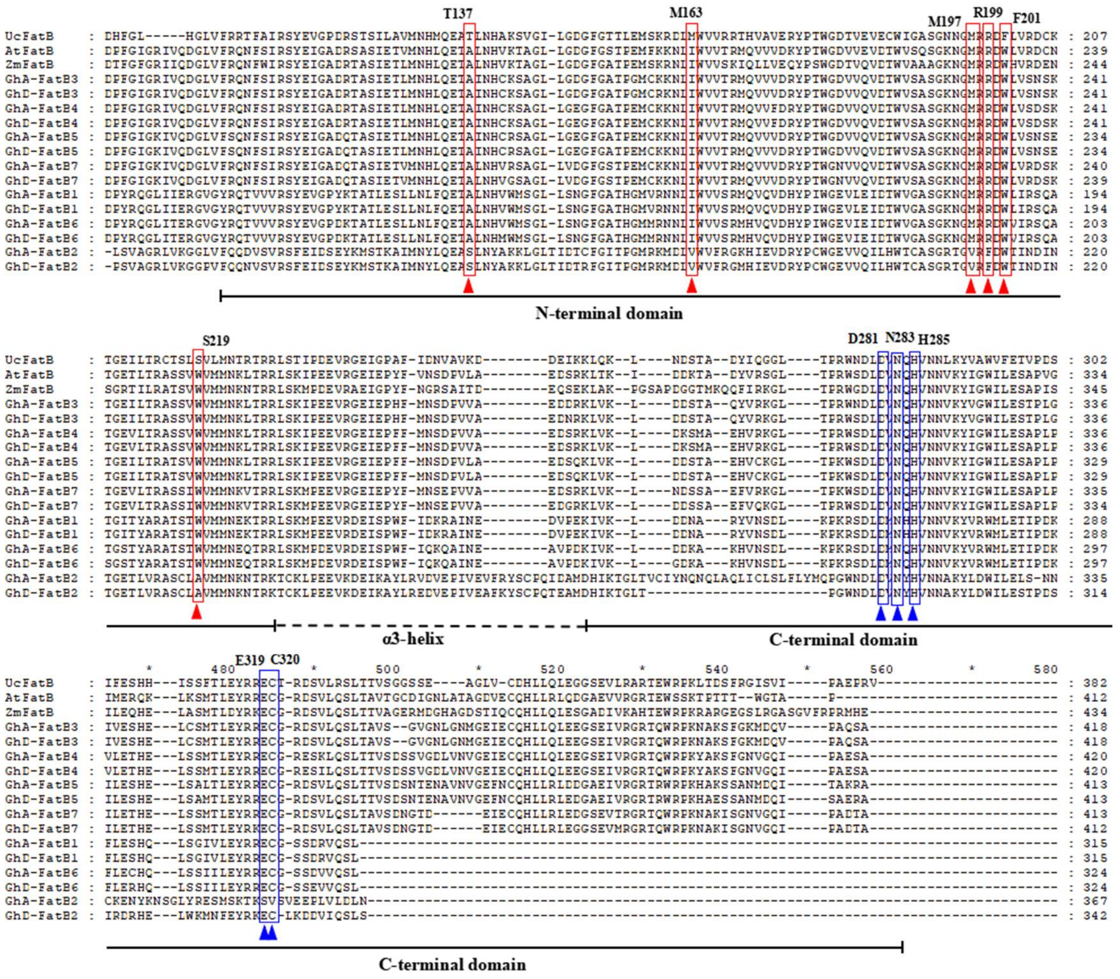

Supplementary Figure S2. Multiple alignments of GhFatB family members. 12:0-ACP specific UcFatB and 16:0-ACP specific tepmples (AtFatB and ZmFatB) are used in this analysis. Red and blue colors represent key amino acids located in substrate binding cavity and catalytic center, respectively.
